# Biosimilars; a unique opportunity for Iran national health sector and national pharmaceutical industry

**DOI:** 10.1186/2008-2231-20-35

**Published:** 2012-09-10

**Authors:** Abdol Majid Cheraghali

**Affiliations:** 1 Department of Pharmacology, University of Baqiyatallah Medical Sciences, Tehran, Iran

**Keywords:** Biopharmaceuticals, Biosimilars, Iran

## 

In 1980s, medicines produced through recombinant DNA technology created a novel opportunity for management of several debilitating and life threatening diseases. However, these medicines are very expensive and therefore not affordable for many patients especially those living in low resourced countries. Biopharmaceuticals mostly have a high unit cost and often prescribed for chronic medical conditions with possibility of long-term use. Therefore, they will impose a burden on either national health care systems or patients’ out of pocket. It is estimated that the average daily treatment of a patient with branded biopharmaceuticals will cost at least 22 times of those for small molecule medicines [1]. Biopharmaceuticals are medicines produced from living organisms via genetic manipulation. Although use of living organisms for production of vaccines has a long history, the first DNA recombinant medicine for human use, human insulin, was approved in the USA in 1982. However, introduction of other biopharmaceuticals including monoclonal antibodies into market later on experienced a much faster pace. Biopharmaceuticals are large and complex molecules which their active substances are mostly polypeptides, glycoproteins, proteins, and nucleic acids. Therefore, it is practically impossible to manufacture an “identical” copy for these molecules. Blood coagulating factors, erythropoietins, gonadotrophins, granulocyte colony stimulating factor (GCSF), human growth hormones (GH), interferons (INF), interleukins and monoclonal antibodies are among the most important marketed biopharmaceuticals in past decades [2].

Despite complexity of their molecules, biopharmaceuticals are very well characterized both for their clinical use and production methods. Biopharmaceuticals are also very sensitive to their production procedures. When the manufacturing process is modified by scaling up or transferring it to alternative facilities, new products might show modified specifications. Even though these manufacturing changes are tightly regulated most of the time they still will cause observable changes in the final product. However, all of these products will be marketed under one single brand name indicating that regulators consider these changes in the range of “highly similar” products. The fact that their administration provides expected clinical outcomes also confirm their comparability.

“Biosimilars” are biopharmaceuticals which are manufactured by non originator pharmaceutical companies following expiration of patent period. According to current guidelines and regulations for granting marketing authorization “similarity” should be proven between biosimilar and its corresponding originator biopharmaceutical.

Biopharmaceuticals have a very fast growing market. It is reported that 32% of products in development pipeline and 7.5% of marketed medicines are biopharmaceuticals which account for around 10% of pharmaceutical expenditure [3]. It is forecasted that by 2020, biopharmaceuticals will sell around US$23 billion in the EU and US$29 billion in the USA and of course all of this market could be challenged by biosimilars [3]. The biopharmaceutical market has expanded drastically in the past two decades. Although most of these medicines enjoyed exclusivity in the market due to their patent; recent years have witnessed expiration of their patent.

## Regulation of biosimilars

Since in coming years more and more biopharmaceuticals will lose their patent protection, many pharmaceutical companies including biotechnology industry and generics manufacturers, as well as regulatory agencies, are becoming increasingly interested in biosimilars [4].

However, contrary to the small molecule medicines generics replication of biopharmaceuticals is somehow a complex and controversy issue. Biopharmaceuticals usually have large and complex molecular structures of protein nature which in many cases replication of these systems in order to produce “identical and similar” molecules are very difficult. Most of the times, small changes in the structure of the final molecule might create different safety and efficacy profile. Therefore changes in the manufacturing process can cause complications for potential biosimilars. New molecules could cause severe immunogenicity reactions. That is why evaluation of biosimilars becomes a serious challenge for both the scientific community and regulatory agencies [5].

Despite presence of biosimilars in some markets since years ago several highly regulated markets such as USA, until recently did not have any regulations for registration of these medicines. In 2004, European Medicine Agency (EMA) was the first well established regulatory authority to develop a comprehensive guideline for dealing with biosimilars [6]. The main concept of “biosimilarity” in this guideline relies on a head to head demonstration of similarity of the new medicine from both physicochemical and biological activity point of view to a reference originator biopharmaceutical. Since then EMA has authorized more than 14 biosimilars for marketing in EU. Until 2010, the FDA lacked the legal authority to approve biosimilar medicines similar to what is already happening in EU. However, with new reform of health care system in USA, FDA has now the authority to review and approve “highly similar” biosimilar medicines.

WHO has published its first guideline for evaluation of biosimilars in 2010 [7,8]. This guideline also relies on a head to head demonstration of biosimilarity of a biosimilar with a registered biopharmaceutical. This head to head comparison involves both quality non-clinical and clinical aspects of the products. Clinical study should design in a way that would be able to demonstrate comparable safety and efficacy between biosimilar and reference biopharmaceutical.

However, there are some experts who believe that performing head to head preclinical and clinical trials between brand and biosimilar may not be necessary and in fact this might deprive patients of cost effectiveness treatment and compromise patients’ affordability [9]. Some other researchers even believe that the clinical trials required by EMA to compare biosimilars and their corresponding originator product may even be a barrier for the development of future, more advanced biopharmaceuticals [10].

Highly regulated markets such as USA and EU have specific requirements for biosimilars regarding comparative studies proofing similar safety, efficacy, purity and potency for biosimilars and their brand comparator. Therefore developing biosimilars in these markets require substantial investments estimated in the range of 75–250 million USD [10]. However, this might not be the case for marketing of biosimilars in less regulated markets. Therefore biosimilars in these markets could have different definitions. Although such products might have acceptable safety and efficacy profile based on local requirements they are not “biosimilars” as they have been defined by FDA, EMA or even WHO guidelines. As an example according to a published report, among several recombinant erythropoietins manufactured in Asia none was comparable with the originator product from structural point of view [11]. However, according to the similar methodology, biosimilar erythropoietin marketed in EU was identical with originator [12]. Therefore there is a very low chance for biosimilars developed outside of EU or USA to be approved by FDA or EMA as biosimilar medicine.

Although in many cases, following any change made in production process of biopharmaceuticals originators should ask for regulatory permission for marketing of their products, authorities even in EU or USA do not ask for new preclinical or clinical data in these cases. If the product fall within the variability of the originator molecule after manufacturing change then the new product would be considered “highly similar” to the originator molecule.

Surprisingly, FDA has recently used a non-clinical based approach for granting marketing authorization to the biosimilars. In 2010, FDA approved a generic Enoxaparin as a fully substitutable generic to Sanofi-Aventis Lovenox (Enoxaparin). In reviewing the Enoxaparin file, FDA used five criteria to establish “sameness” for these two products. None of them were pre-clinical or clinical data. In fact FDA used the analytical characterization to grant similarity status to the generic Enoxaparin [10].

## Cost impact of biosimilars

Although global sale of biopharmaceuticals in 2009 was over 93 billion USD, their sales expected to grow at least as twice as fast compared to those of small molecules. It is estimated that by 2016, ten of the 20 top selling medicines will be biological medicines. It is also estimated that share of off patent products in biopharmaceutical market will exceed 40% of global sale in 2015 [3,13]. This optimistic market forecast for biopharmaceuticals has convinced both multinational and national pharmaceuticals to allocate substantial R&D resources on developing these medicines.

Cost containment pressures in healthcare systems could be considered as a key driver for the biosimilars market. Current biopharmaceuticals on the market are too expensive that even a modest price reduction through marketing of biosimilars could attract attention of policy makers in health sector. Marketing of biosimilars not only will facilitate access to these highly effective medicines, but also reported to be cost effective treatments and could create substantial saving for national health sectors [13]. It has been reported that newly established biosimilar medicines already generated around 1.4 billion Euros saving per year for European healthcare systems [14]. Obviously this saving might be used for next generation of originator medicines. Therefore, biosimilars could also have a major impact on affordability of biopharmaceuticals in all markets and especially in low resourced countries.

Biosimilars market is so lucrative that both multinational pharmaceutical companies and local industries have rushed for manufacturing such medicines. For example in 2011 Sandoz announced that it was starting a phase II clinical trial for a biosimilar version of Rituximab. Sandoz has already several biosimilars either on the market or at various stages of development [15]. In other countries, even governments decided to support manufacturing of the biosimilars. South Korean government is actively promoting the biosimilars industry in order to make South Korea a market leader in this field. The government is providing both financial and institutional support and is aiming to take a 22% share of the global market by 2020 [16]. Some Indian and Chinese companies also decided to invest on biosimilars. Their main aim is to manufacture biosimilars which are much cheaper than those produced by other companies. Cipla and a Chinese partner, BioMab, investing 124 million Euros to build plants in India and China to produce biopharmaceuticals.

## Biopharmaceuticals in Iran market

Iran national pharmaceutical industry has a more than half century history. However, these are mostly generic based companies which produce small molecule medicines. Since decade ago some newly established science based Iranian pharmaceutical companies started projects on developing biopharmaceutical. Iran government has also allocated substantial resources for supporting local pharmaceutical companies to manufacture biopharmaceuticals. This include both financial and administration supports. Pharmaceutical companies in countries such as Iran do not have access to the production procedures of originators including cell type, fermentation and purification procedures. Therefore they could not claim “similarity” for their products to those of originator brand.

Despite the fact that biopharmaceuticals which were produced by local Iranian industry in past decade including INFs, GCSF and GH have received marketing authorization for local market, none of them received comprehensive evaluation according to those of internationally recognized guidelines for biosimilars. Registration of these biopharmaceuticals has mainly followed registration path for “biogeneric” medicines and their application for marketing authorization handled based on case by case. Since 2003 about 6 biopharmaceuticals produced as non originator copy have been registered by Iran national authority. These include erythropoietins, INFs, GH and GCSF. Another 16 are in pipeline and expected to reach Iran market with similar approach in coming years [17].

Iran has a fairly well established national Adverse Drug Reaction (ADR) reporting system and so far no serious or unexpected ADRs related to administration of these locally manufactured biopharmaceuticals have been reported to the national health authorities. This could be considered as an indication of safety of these locally manufactured biopharmaceuticals.

However, based on a national guideline which was mainly adapted from WHO guideline, since 2006 performing a double blind controlled clinical trial with small sample size for locally manufactured biopharmaceuticals becomes obligatory. Although Iran national regulatory authority (NRA) has tried to use WHO guideline on biosimilars for granting marketing authorization, there are clearly differences between WHO guideline and current Iran national guideline for registration of locally produced biopharmaceuticals [17].

Iran is not a member of World Trade Organization (WTO) and therefore local pharmaceutical industries have the privilege not to respect internationally recognized intellectual property rights and patents regarding medicines including biopharmaceuticals. Although there are national laws and regulations for protection of patents and brands registered by Iranian companies inside the Iran territory, these regulations do not apply for foreign companies and their products registered outside of Iran. Therefore the tension between intellectual property rights and competition law, which can be seen in many industries, is not a valid concern for local pharmaceutical industry in Iran. Although Iran has applied for WTO membership, due to current local and international political situations such membership will not happen in near future. Therefore it cannot be considered as an eminent concern for manufacturing of patent biopharmaceuticals by local industry.

Providing that local pharmaceutical companies could have access to the production procedures of biopharmaceuticals, they would be able to manufacture both patent and off patent biopharmaceuticals. Although conventional local pharmaceutical companies do not have the capacity to provide high tech biopharmaceuticals [18-20], newly established science based Iranian pharmaceutical companies have shown the ability to access to the production procedures of biopharmaceuticals and actually manufacture such medicines.

## Conclusion

Health care expenditure has been raised drastically in recent years. Costs attributed to the medicines and especially biopharmaceuticals consumes substantial share of healthcare budget. Therefore health services providers including healthcare authorities or insurance companies are struggling to find ways to reduce the healthcare budget. Although biosimilars proved to be a cost effective intervention for providing effective treatment for patients, regulatory approaches proposed for marketing of these medicines by organizations such as EMA, FDA or even WHO may not suite needs of Iran market.

Iran authorities should keep in mind that WHO biosimilar guideline is mainly adapted from EMA guideline and there is no proof that this guideline is in fact in line with the needs and interests of national pharmaceutical markets in low resourced countries such as Iran. Iran national pharmacovigilance center proved to be an efficient center. Therefore in order to make a balance between regulation of biopharmaceuticals manufactured by local industry and affordability of these medicines, Iran NRA should use this capacity as a tool in regulation of locally manufactured biopharmaceuticals. Author believes that Iran NRA should accept pharmacokinetic and pharmacodynamic equivalence between the originator and locally manufactured biopharmaceuticals as a measure of clinical comparability. However, biopharmaceuticals have varying potentials for immunogenicity that could be changed based on number of factors, including manufacturing processes. Therefore, it is essential that rigorous pharmacovigilance approach should be maintained in order to exclude possible immunogenicity or other important adverse events related to the locally produced biopharmaceuticals.
